# Ferromagnetism and optical properties of La_1 − *x*_Al_*x*_FeO_3_ nanopowders

**DOI:** 10.1186/1556-276X-9-498

**Published:** 2014-09-15

**Authors:** Yutana Janbutrach, Sitchai Hunpratub, Ekaphan Swatsitang

**Affiliations:** 1 Materials Science and Nanotechnology Program, Faculty of Science, Khon Kaen University, Khon Kaen 40002, Thailand; 2 Integrated Nanotechnology Research Center and Department of Physics, Faculty of Science, Khon Kaen University, Khon Kaen 40002, Thailand; 3 Nanotec-KKU Center of Excellence on Advanced Nanomaterials for Energy Production and Storage, Khon Kaen 40002, Thailand

**Keywords:** Ferromagnetism, Optical properties, Polymerization complex method, La_1 − *x*_ Al_*x*_FeO_3_, Nanopowders

## Abstract

La_1 − *x*_Al_*x*_FeO_3_ (*x* = 0.0, 0.05, 0.1, 0.2, 0.3, 0.4, and 0.5) nanopowders were prepared by polymerization complex method. All prepared samples were characterized by X-ray diffraction (XRD), scanning electron microscopy (SEM), transmission electron microscopy (TEM), Fourier transform infrared spectroscopy (FT-IR), and UV-vis spectrophotometry (UV-vis). The magnetic properties were investigated using a vibrating sample magnetometer (VSM). The X-ray results of all samples show the formation of an orthorhombic phase with the second phase of α-Fe_2_O_3_ in doped samples. The crystallite sizes of nanoparticles decreased with increasing Al content, and they are found to be in the range of 58.45 ± 5.90 to 15.58 ± 4.64 nm. SEM and TEM images show the agglomeration of nanoparticles with average particle size in the range of 60 to 75 nm. The FT-IR spectra confirm the presence of metal oxygen bonds of O-Fe-O and Fe-O in the FeO_6_ octahedra. The UV-vis spectra show strong absorption peaks at approximately 285 nm, and the calculated optical band gaps are found to be in the range of 2.05 to 2.09 eV with increasing Al content. The M-H loop of the pure sample is antiferromagnetic, whereas those of the doped samples tend to be ferromagnetic with increasing Al content. The magnetization, remanent magnetization, and coercive field of the Al-doped sample with *x* = 0.5 are enhanced to 1.665 emu/g, 0.623 emu/g, and 4,087.0 Oe, respectively.

## Background

LaFeO_3_ with an orthorhombic phase of the ABO_3_-type perovskite structure has become a currently attractive research topic because it is proposed for various applications in several advanced technologies such as catalysts [1-3], various kinds of chemical and gas sensors [4-9], and electrode materials in solid oxide fuel cells [10]. In general, LaFeO_3_ consists of FeO_6_ octahedral units with La^3+^ ions at the corners [11,12]. The advantage of this structure is the replaceability of metallic ions at both A and B sites by various transition metals. Pure and doped LaFeO_3_ (Pd, Al, Zn, Ag, Sr, Ir, Ca, Co, etc.) were studied for various purposes and aspects with reports of optical, electrical, and magnetic properties [13-25].

Research on pure and doped LaFeO_3_ nanostructures reveal that the property and quality of the materials are strongly influenced by the synthesis method. The synthesis method is usually related to the specific preparation conditions which can result in various properties of the end products. Various techniques were employed for the synthesis of pure and doped LaFeO_3_ such as sol-gel/combustion method [26-40], microwave-assisted method [41-43], solid-state reaction method [14,44-46], thermal decomposition [47,48], microemulsion method [49], hydrothermal method [50-52], hot soap method [53], spray drying [54], electrospinning [55], drip pyrolysis [19], and polymerization complex method [56-59]. However, polymerization complex method based on polyesterification between citric acid (CA) and ethylene glycol (EG) is the most attractive because it is simple, cost effective, time saving, and environmentally benign.

Thus, we propose in this research the synthesis of La_1 − *x*_Al_*x*_FeO_3_ (*x* = 0, 0.05, 0.1, 0.2, 0.3, 0.4, and 0.5) nanopowders using a simple polymerization complex method. The magnetic and optical properties of the products were studied. The magnetization, coercive field, and remanent magnetization are measured, and they are expected to be enhanced due to the substitution of small-radius ions of Al on the La site.

## Methods

La_1 − *x*_Al_*x*_FeO_3_ (*x* = 0, 0.05, 0.1, 0.2, 0.3, 0.4, and 0.5) were synthesized by polymerization complex method. Stoichiometric amounts of iron nitrate (Fe(NO_3_)_3_.9H_2_O, Kanto Chemical Co., Chuo-ku, Japan, 99.9%), lanthanum nitrate (LaN_3_O_9_.6H_2_O, Fluka, Seelze, Germany, 99.0%), and aluminum nitrate (Al(NO_3_)_3_.9H_2_O, Carlo Erba Reagenti, Milan, Italy, 99.0%) in the ratio of 1 – *x*:*x*:1 (La:Al:Fe) with 1 g of citric acid (C_6_H_8_O_7_.H_2_O, VWR International Ltd., Radnor, PA, USA, 99.7%) were dissolved in 40 mL ethylene glycol and 20 mL deionized (DI) water. The mixture was magnetically stirred for 1 h in order to obtain stable metal-citric acid complexes. The obtained solution was continuously stirred at 70°C for 1 h. This solution was dried at 120°C on a hot plate. The obtained powders were pre-calcined at 400°C for 3 h to burn out the polymer. The pre-calcined powders were ground and further calcined at 900°C for 3 h in air.

The calcined powders were characterized using an X-ray diffractometer (XRD; XRD-6100, Shimadzu, Kyoto, Japan) with CuKα_1_ radiation (*λ* = 1.5405 Å). The morphologies of the synthesized products were observed using a scanning electron microscope (SEM; 1450VP, LEO, Hurley, UK) and a transmission electron microscope (TEM; Tecnai G2 20, FEI, Hillsboro, OR, USA). The components of the powders were analyzed by energy-dispersive X-ray spectroscopy (EDX; Tecnai G2 20, FEI). Fourier transform infrared spectroscopy (FT-IR; Spectrum One FT-IR, Perkin Elmer Instrument, Waltham, MA, USA) was employed to investigate functional groups in all samples. The optical properties were studied by ultraviolet-visible spectroscopy (UV-vis; UV-3101PC, Shimadzu). The magnetizations of all samples were measured using a vibrating sample magnetometer (VSM; VersaLab™ Cryogen-free, Quantum Design, San Diego, CA, USA).

## Results and discussion

### XRD analysis

**Figure 1:**
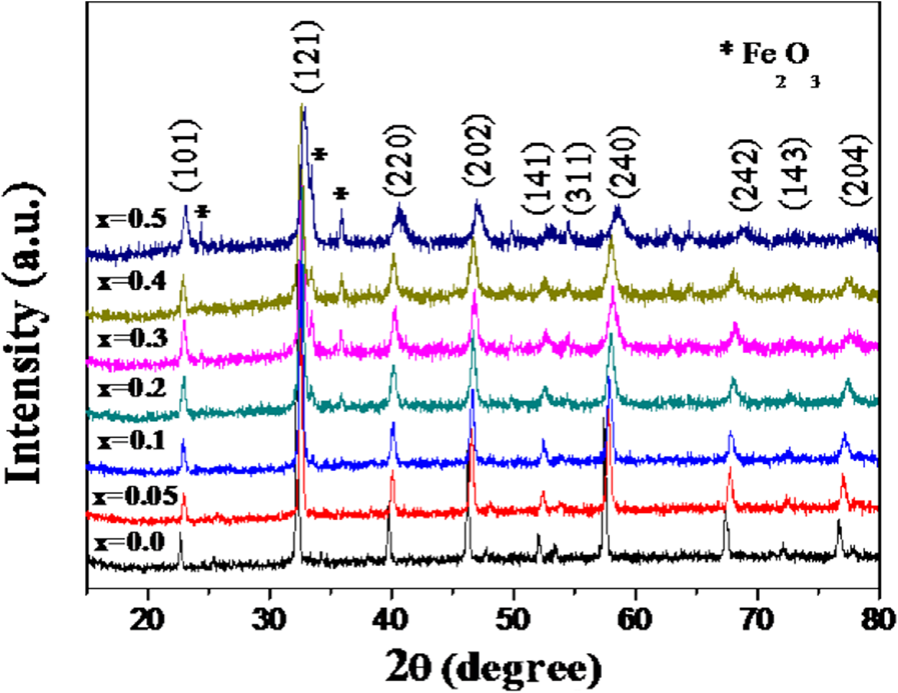
**XRD patterns of La**_**1 − *x***_**Al**_***x***_**FeO**_**3 **_**(*x*** **= 0.0, 0.05, 0.1, 0.2, 0.3, 0.4, and 0.5) nanopowders.**

The XRD patterns of La_1 − *x*_Al_*x*_FeO_3_ (*x* = 0, 0.05, 0.1, 0.2, 0.3, 0.4, and 0.5) nanopowders are shown in Figure 1. The results indicate that the products are a perovskite oxide of an orthorhombic structure with the second phase of α-Fe_2_O_3_ in the doped samples of *x* = 0.2 to 0.5. The XRD results are in good agreement with the standard data of LaFeO_3_ (JCPDS card no: 37-1493) and α-Fe_2_O_3_ (JCPDS card no: 89-0595). The average crystallite size is determined from the X-ray line broadening of the (101), (121), (220), (202), (240), (242), and (204) diffraction peaks using the Scherrer equation, and it is found to be decreased with increasing Al content, as summarized in Table 1. The lattice parameters *a*, *b*, and *c* of the doped samples decreased with the increase of Al content due to the replacement of the larger La^3+^ ion (radius approximately 1.36 Å) by a smaller Al^3+^ ion (radius approximately 0.535 Å) [22], as summarized in Table 1. The significant change in the decrease of lattice parameters with increasing Al content is confirmed by the shift of the diffraction peaks to a higher diffraction angle. On the other hand, Al^3+^ ions can be substituted on B sites of Fe^3+^ ions because the ionic radius of Al^3+^ is close to that of the Fe^3+^ ion (radius approximately 0.78 Å), resulting in the formation of the impurity phase of α-Fe_2_O_3_.

**Table 1:** **Lattice parameter and crystallite size of La**_**1 − *x***_**Al**_***x***_**FeO**_**3 **_**nanopowders**

**La**_**1 − *x***_**Al**_***x***_**FeO**_**3**_	**Lattice parameter (Å)**			**Average crystallite size (Å)**
	***a***	***b***	***c***	
*x* = 0.0	5.559	7.862	5.560	58.45 ± 5.90
*x* = 0.05	5.544	7.848	5.549	39.00 ± 1.03
*x* = 0.1	5.536	7.834	5.539	29.83 ± 7.84
*x* = 0.2	5.503	7.812	5.522	24.30 ± 3.76
*x* = 0.3	5.503	7.790	5.506	23.23 ± 5.22
*x* = 0.4	5.506	7.785	5.509	22.35 ± 4.77
*x* = 0.5	5.443	7.762	5.502	15.58 ± 4.64

### SEM analysis

**Figure 2:**
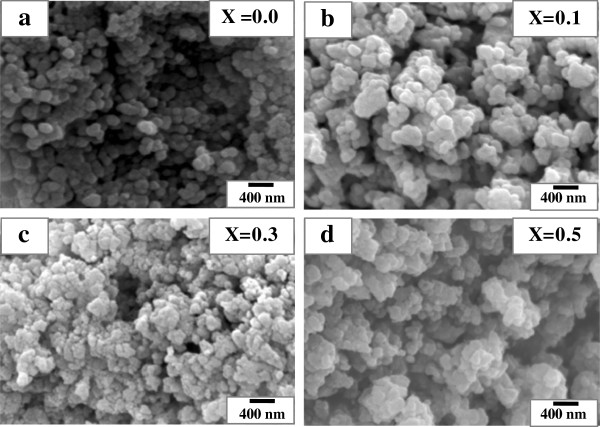
**SEM micrographs of La**_**1 − *x***_**Al**_***x***_**FeO**_**3 **_**nanopowders. (a)***x* = 0.0. **(b)***x* = 0.1. **(c)***x* = 0.3. **(d)***x* = 0.5.

The SEM micrographs of La_1 − *x*_Al_*x*_FeO_3_ (*x* = 0.0, 0.1, 0.3, and 0.5) nanopowders are shown in Figure 2. In Figure 2a, the powders are almost irregularly nanoagglomerated with a mean size of approximately 60 to 75 nm. In Figure 2b,c,d, agglomeration of nanoparticles with a size larger than 100 nm and grain growth can be observed in doped samples. Moreover, the SEM images reveal a uniform grain size distribution and homogeneous nanostructure.

### TEM analysis

**Figure 3:**
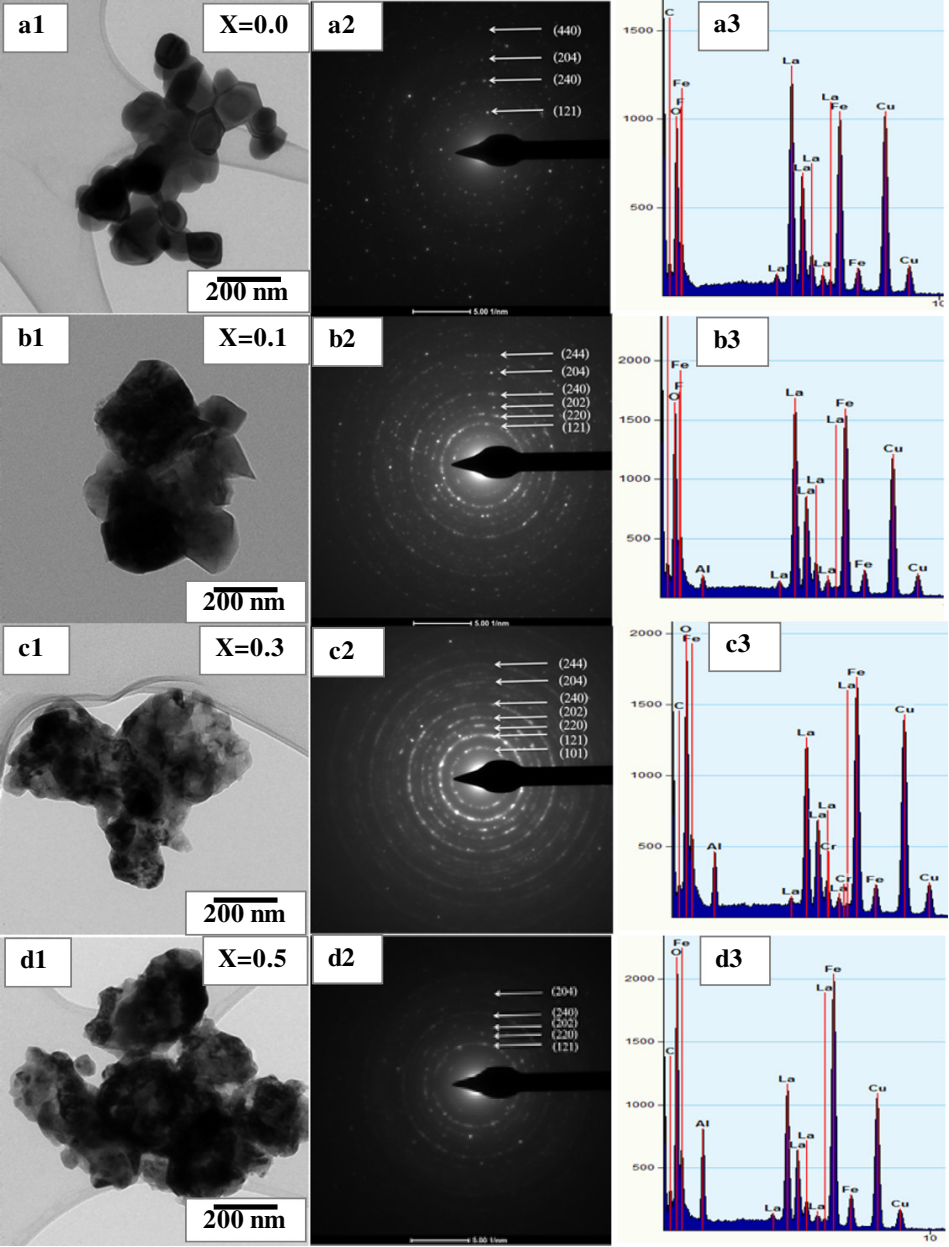
**Bright-field TEM images (a1-d1) with the corresponding SAED patterns (a2-d2) and EDX spectra (a3-d3) of La**_**1 − *x***_**Al**_***x***_**FeO**_**3 **_**nanopowders. (a)***x* = 0.0. **(b)***x* = 0.1. **(c)***x* = 0.3. **(d)***x* = 0.5.

Figure 3a,b,c,d shows bright-field TEM images with the corresponding selected area electron diffraction (SAED) patterns and EDX spectra of La_1 − *x*_Al_*x*_FeO_3_ (*x* = 0.0, 0.1, 0.3, and 0.5) nanopowders. It is obvious in Figure 3a1,b1,c1,d1 that the particulates consist of the agglomeration of numerous nanocrystallite particles of irregular shape, corresponding to the SEM observation in Figure 2. The average particle size is estimated and found to be approximately 60 to 75 nm. The SAED patterns in Figure 3a2,b2,c2,d2 show ring patterns, indicating that all doped samples are polycrystalline. Each SAED pattern can be indexed to a certain crystalline plane which is found to be consistent with that of the XRD results in Figure 1. The EDX spectra of these samples are shown in Figure 3a3,b3,c3,d3. The EDX results clearly show that all samples contain La, Fe, Al, and O with higher intensity peaks of Al in samples of high Al content. The Cu peaks that appeared come from the copper grid.

### FT-IR analysis

**Figure 4:**
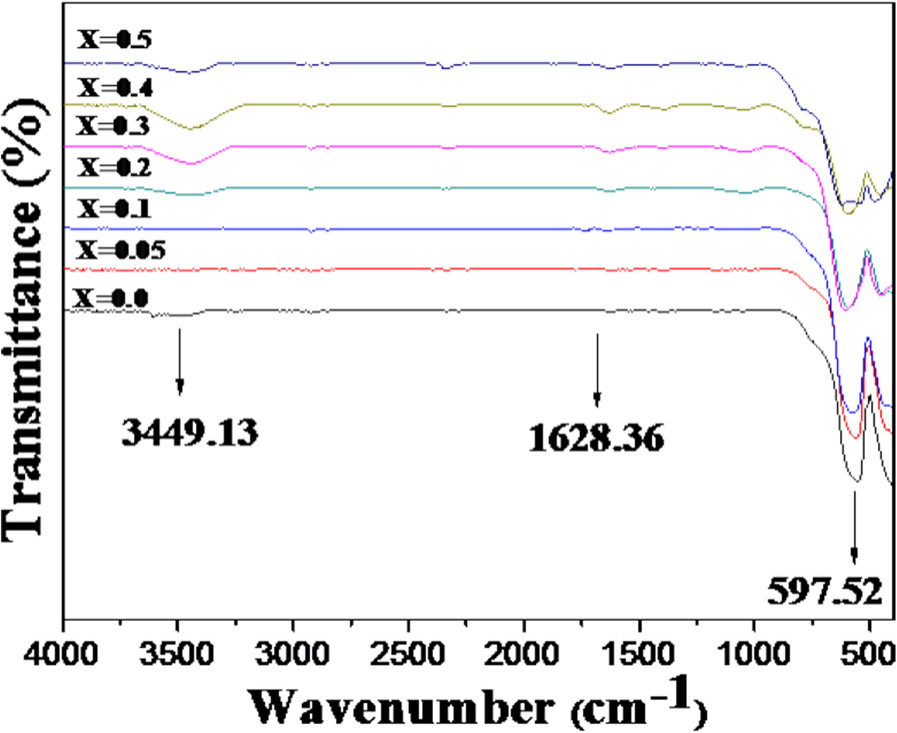
**FT-IR spectra of La**_**1 − *x***_**Al**_***x***_**FeO**_**3 **_**(*x*** **= 0.0, 0.05, 0.1, 0.2, 0.3, 0.4, and 0.5) nanopowders.**

Figure 4 shows the FT-IR spectra of La_1 − *x*_Al_*x*_FeO_3_ (*x* = 0, 0.05, 0.1, 0.2, 0.3, 0.4, and 0.5) nanopowders. All spectra show broad absorption peaks at approximately 3,449.13 cm^−1^, corresponding to the symmetric and asymmetric stretching modes of water molecules. The observed broad band at approximately 1,600 cm^−1^ corresponds to the bending mode of O-H bond. The strong absorption peaks in the range of 500 to 600 cm^−1^ reveal the presence of metal oxygen bonds which can be assigned to the vibrations of Fe-O and O-Fe-O bonding in the octahedral structure of La_1 − *x*_Al_*x*_FeO_3_. These results are in good agreement with the FT-IR spectra of pure and doped LaFeO_3_ reported in the literature [14,41,43,47,50].

### UV-vis analysis

**Figure 5:**
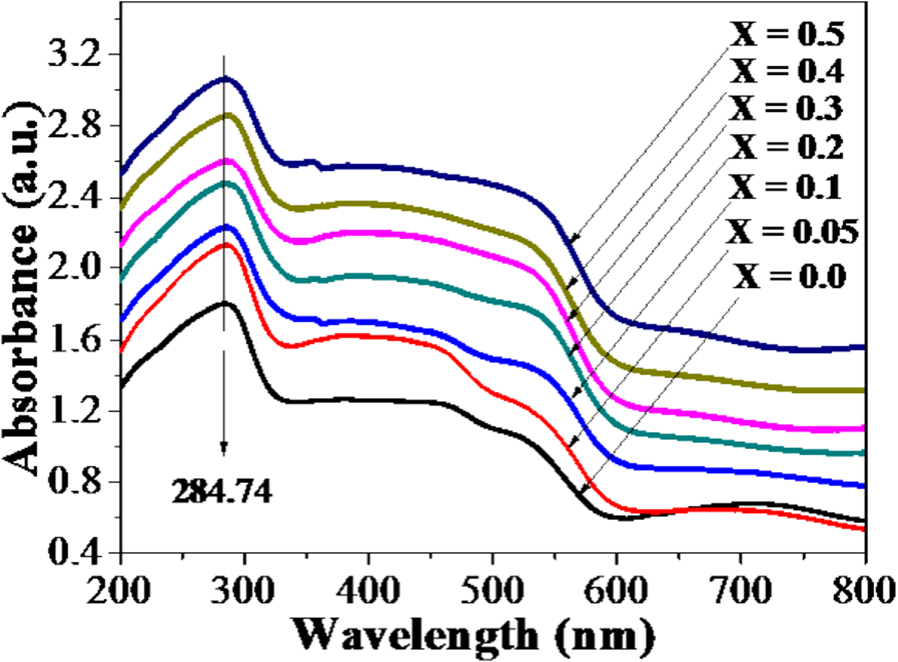
**UV-vis spectra of La**_**1 − *x***_**Al**_***x***_**FeO**_**3 **_**(*x*** **= 0.0, 0.05, 0.1, 0.2, 0.3, 0.4, and 0.5) nanopowders.**

**Figure 6:**
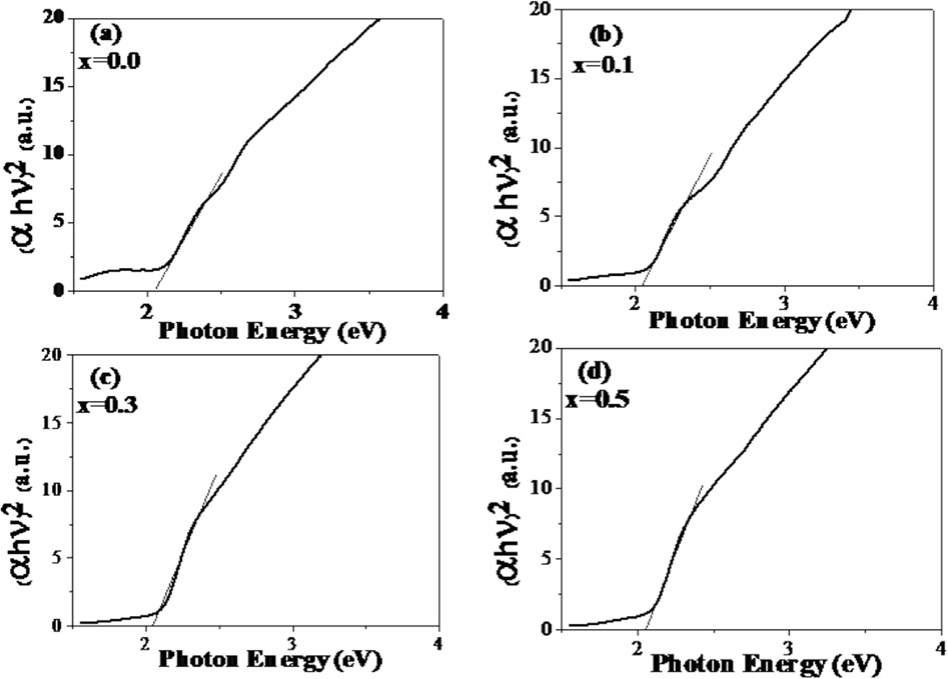
**Plots of ( *αhν *)**^**2 **^**as a function of photon energy of La**_**1 − *x***_**Al**_***x***_**FeO**_**3 **_**nanopowders. (a)***x* = 0.0. **(b)***x* = 0.1. **(c)***x* = 0.3. **(d)***x* = 0.5.

The UV-vis spectra of La_1 − *x*_Al_*x*_FeO_3_ (*x* = 0, 0.05, 0.1, 0.2, 0.3, 0.4, and 0.5) nanopowders are shown in Figure 5. In Figure 5, broad absorption peaks are observed in all samples at approximately 285 nm with the infinitesimal redshifted to approximately 290 nm. From the plot of (*αhν*)^2^ vs. *hν* in Figure 6a,b,c,d, the optical band gaps (*E*_g_) of the samples can be determined by extrapolating the slope to the zero value of (*αhν*)^2^, and the obtained values are summarized in Table 2. It is found that the optical band gaps do not significantly vary with increasing Al content.

**Table 2:** **Coercive field (*H***_**c**_**), magnetization ( *M *), remanent magnetization (*M***_**r**_**), and optical band gap (*E***_**g**_**) of La**_**1 − *x***_**Al**_***x***_**FeO**_**3 **_**nanopowders**

**La**_**1 − *x***_**Al**_***x***_**FeO**_**3**_	***H***_**c **_**(Oe)**	***M *** **(emu/g)**	***M***_**r **_**(emu/g)**	***E***_**g **_**(eV)**
*x* = 0.0	366.8	0.202	0.007	2.05
*x* = 0.05	591.2	0.291	0.008	2.07
x = 0.1	1,597.2	0.196	0.012	2.07
*x* = 0.2	3,390.6	0.300	0.038	2.07
*x* = 0.3	5,308.4	0.509	0.158	2.09
*x* = 0.4	4,399.3	0.899	0.301	2.09
*x* = 0.5	4,087.0	1.665	0.623	2.07

### VSM analysis

**Figure 7:**
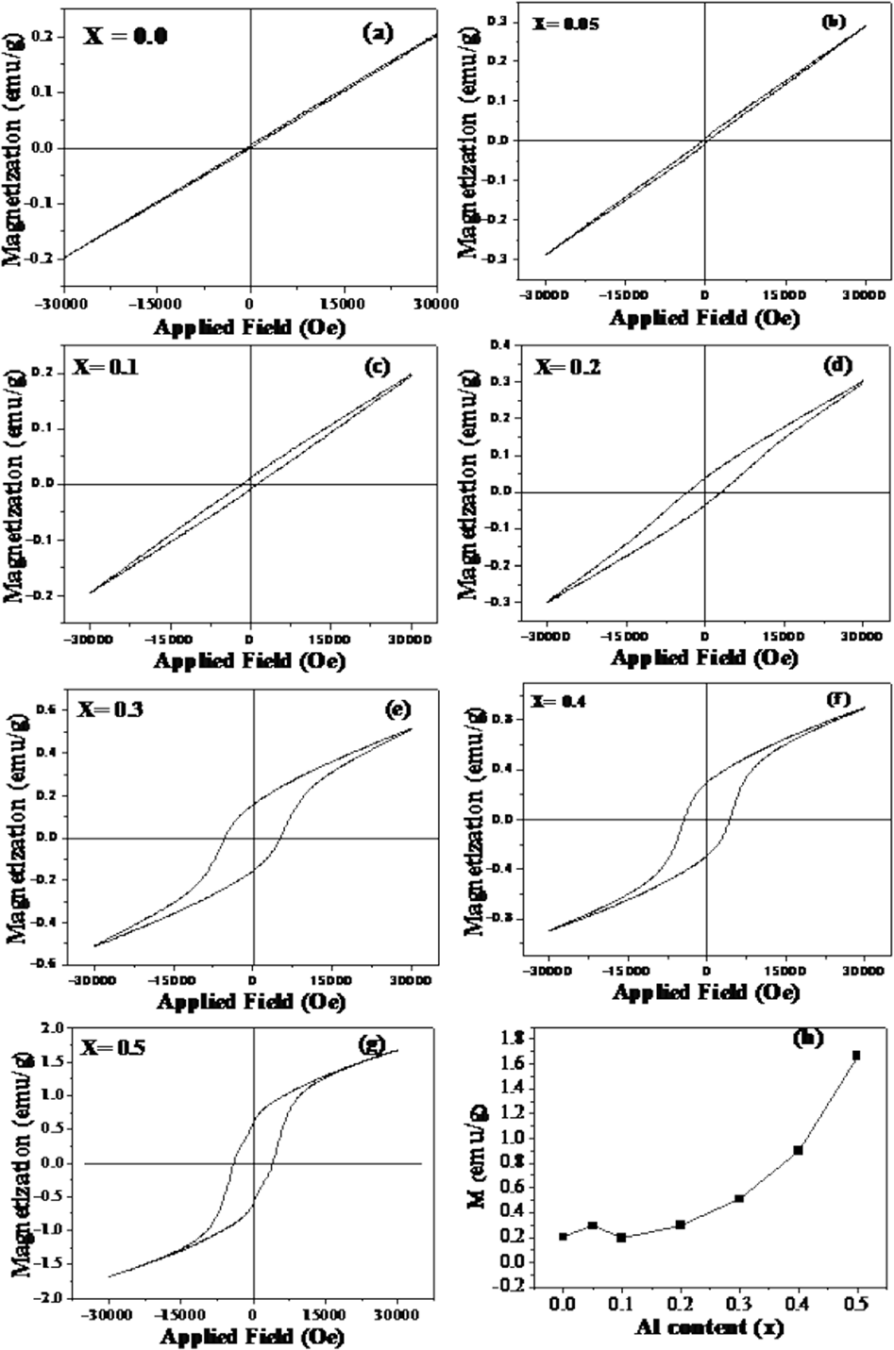
**Magnetization measurements at room temperature of La**_**1 − *x***_**Al**_***x***_**FeO**_**3 **_**nanopowders. (a)***x* = 0.0. **(b)***x* = 0.05. **(c)***x* = 0.1. **(d)***x* = 0.2. **(e)***x* = 0.3. **(f)***x* = 0.4. **(g)***x* = 0.5. **(h)***M* as a function of Al content.

**Figure 8:**
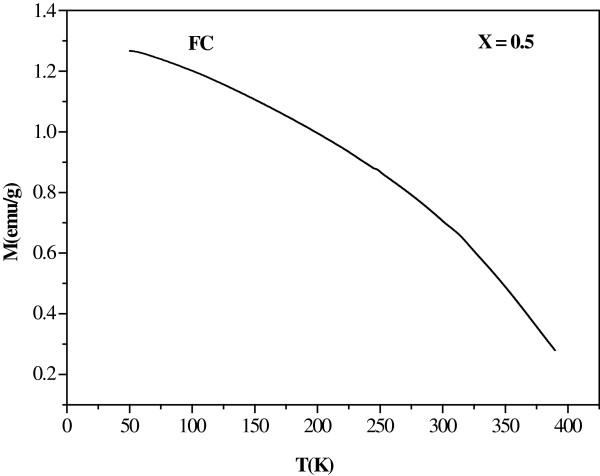
**Magnetization of La**_**0.5**_**Al**_**0.5**_**FeO**_**3 **_**nanopowder as a function of temperature measured by field cooling process.**

Figure 7a,b,c,d,e,f,g shows the magnetization curves of La_1 − *x*_Al_*x*_FeO_3_ (*x* = 0, 0.05, 0.1, 0.2, 0.3, 0.4, and 0.5) nanopowders measured at room temperature by VSM. As can be seen in Figure 7a, the magnetization curve of the pure sample is very narrow, indicating the antiferromagnetic behavior of the sample, while those of the doped samples show larger loops of ferromagnetic behavior with higher magnetization according to higher Al content (Figure 7b,c,d,e,f,g). In addition, the values of coercive field (*H*_c_), magnetization (*M*), and remanent magnetization (*M*_r_) are enhanced with increasing Al content, as summarized in Table 2. In general, it is well known that pure LaFeO_3_ exhibits antiferromagnetic behavior. This behavior is due to the anti-alignment of the magnetic moments of the Fe^3+^ ions. However, LaFeO_3_ can behave ferromagnetically due to the small crystallite size. The decrease of crystallite size can increase the uncompensated spins at the surface [60,61]. In our work, it is evident in Table 1 that the crystallite size of La_1 − *x*_Al_*x*_FeO_3_ decreases for higher Al content, resulting in the enhancement of ferromagnetism with higher *M* value. In addition, the second phase of α-Fe_2_O_3_ detected in the XRD measurements may also be attributed to the ferromagnetism in La_1 − *x*_Al_*x*_FeO_3_. Figure 8 shows the temperature-dependent magnetization of La_0.5_Al_0.5_FeO_3_ nanopowder investigated by field-cooled (FC) measurement in the temperature range of 50 to 390 K. The *M* decreases as the temperature increases because of the thermal fluctuations causing the randomization of polarization direction. It is clearly seen in Figure 8 that the zero value of magnetization cannot be observed in the temperature range of measurement, implying that the Curie temperature (*T*_c_) is above 400 K.

## Conclusions

In summary, La_1 − *x*_Al_x_FeO_3_ (*x* = 0, 0.05, 0.1, 0.2, 0.3, 0.4, and 0.5) nanopowders were successfully synthesized by polymerization complex method at a temperature of 900°C for 3 h in air. XRD analysis reveals an orthorhombic phase of the nanopowders with average crystallite size in the range of 15.58 ± 4.64 to 58.54 ± 5.90 nm. The impurity phase of α-Fe_2_O_3_ is found in doped samples of *x* ≥ 0.2. SEM and TEM images show agglomerated nanoparticles of irregular shape with estimated particle sizes in the range of 60 to 75 nm. The lattice parameters are found to decrease with increasing Al content. The EDX results clearly show only the main peaks of La, Fe, Al, and O in all samples. The UV-vis spectra show the infinitesimal shift from 285 to 290 nm as the Al content is increased. The increase of Al content does not significantly affect the optical band gaps which are found to be in the range of 2.05 to 2.09 eV. Al^3+^ substitution in LaFeO_3_ crystals can enhance the magnetization (*M*), coercive field (*H*_c_), and remanent magnetization (*M*_r_) of Al-doped samples by a factor of 8, 11, and 89, respectively. The ferromagnetism in La_1 − *x*_Al_*x*_FeO_3_ is due to the size effect and impurity.

## Competing interests

The authors declare that they have no competing interests.

## Authors' contributions

YJ designed and carried out all the experiments and data analysis and participated in preparing the draft of the manuscript. SH co-supervised the research and gave discussion. ES, the project coordinator, supervised the research, designed the experiment, participated in preparing the draft of the manuscript, and revised the manuscript. All authors read and approved the final manuscript.
